# Congenital proximal radioulnar synostosis treated with derotational osteotomy: a pediatric case report from Ethiopia

**DOI:** 10.1097/RC9.0000000000000806

**Published:** 2026-08-11

**Authors:** Ashenafi Negash Tekle, Dawit Alemayehu Tesfaye, Netsanet Abebe Mengiste, Samuel Workineh Assefa, Mateyas Bizualem Agegnehu, Tesfatsion H Michael Lapiso

**Affiliations:** Department of Orthopedic Surgery and Traumatology, St. Paul’s Hospital Millennium Medical College, Addis Ababa, Ethiopia

**Keywords:** case report, Cleary classification, congenital radioulnar synostosis, derotational osteotomy, Ethiopia, forearm deformity, pediatric orthopedics

## Abstract

**Introduction and importance:**

Congenital proximal radioulnar synostosis is a rare skeletal anomaly causing a fixed forearm position and significant functional disability. We present the first surgically treated case from Ethiopia, demonstrating the efficacy of derotational osteotomy.

**Case presentation:**

A 7-year-old boy presented with fixed pronation of the right forearm since birth, with difficulty feeding himself and writing properly. Radiographs confirmed Cleary-Omer Type IV synostosis. A derotational osteotomy at the synostosis site was performed with Kirschner-wire fixation and long-arm splint immobilization.

**Clinical discussion:**

At the final two-year follow-up, the forearm rested in neutral with a functional rotational arc from 10° of pronation to 60° of supination. The child performs all daily activities independently without compensatory shoulder movements.

**Conclusion:**

Derotational osteotomy with Kirschner-wire fixation proved effective and feasible for congenital proximal radioulnar synostosis, even in settings where advanced implants are unavailable.

## Introduction

Congenital proximal radioulnar synostosis results from failed separation of the radius and ulna during embryogenesis, with an estimated incidence of 1 in 2000–6000 live births^[^1,2^]^. The bony fusion abolishes forearm rotation, and when fixed in severe pronation, children struggle with feeding, hygiene, and writing^[^3^]^. Cleary and Omer’s 1985 classification remains the standard, with Type IV, featuring osseous fusion and anterior radial head dislocation, representing the most functionally limiting variant^[^4^]^.

HIGHLIGHTS

- This is the first documented surgical case of congenital radioulnar synostosis from Ethiopia, thereby addressing a significant gap in African pediatric orthopedic literature.
- Derotational osteotomy using basic Kirschner-wire fixation achieved excellent functional outcomes, demonstrating that sophisticated implants are not prerequisites for successful correction.
- A 7-year-old with severe Type IV synostosis regained near-complete supination and independence in daily activities, transforming his quality of life within 6 months.
- Two-year follow-up without complications validates the safety and durability of this straightforward surgical approach in resource-limited settings.
- The case emphasizes the importance of timely surgical referral for children with fixed forearm deformities, particularly in regions where specialized pediatric orthopedic services remain scarce.

Published African cases remain scarce, with isolated reports from Nigeria and Ghana^[^5,6^]^. We present what appears to be the first published surgically treated case from Ethiopia, demonstrating excellent outcomes using basic fixation techniques. This case report has been reported in line with the SCARE 2025 checklist^[^7^]^.

## Case presentation

A 7-year-old right-hand-dominant boy presented to a tertiary referral hospital in Ethiopia with a right forearm deformity noticed during infancy. The family sought care when school-age functional limitations became unmanageable; the child had difficulty feeding himself, required help washing his face, and compensated by positioning his entire body sideways when writing (Table 1).

**Figure 1.**
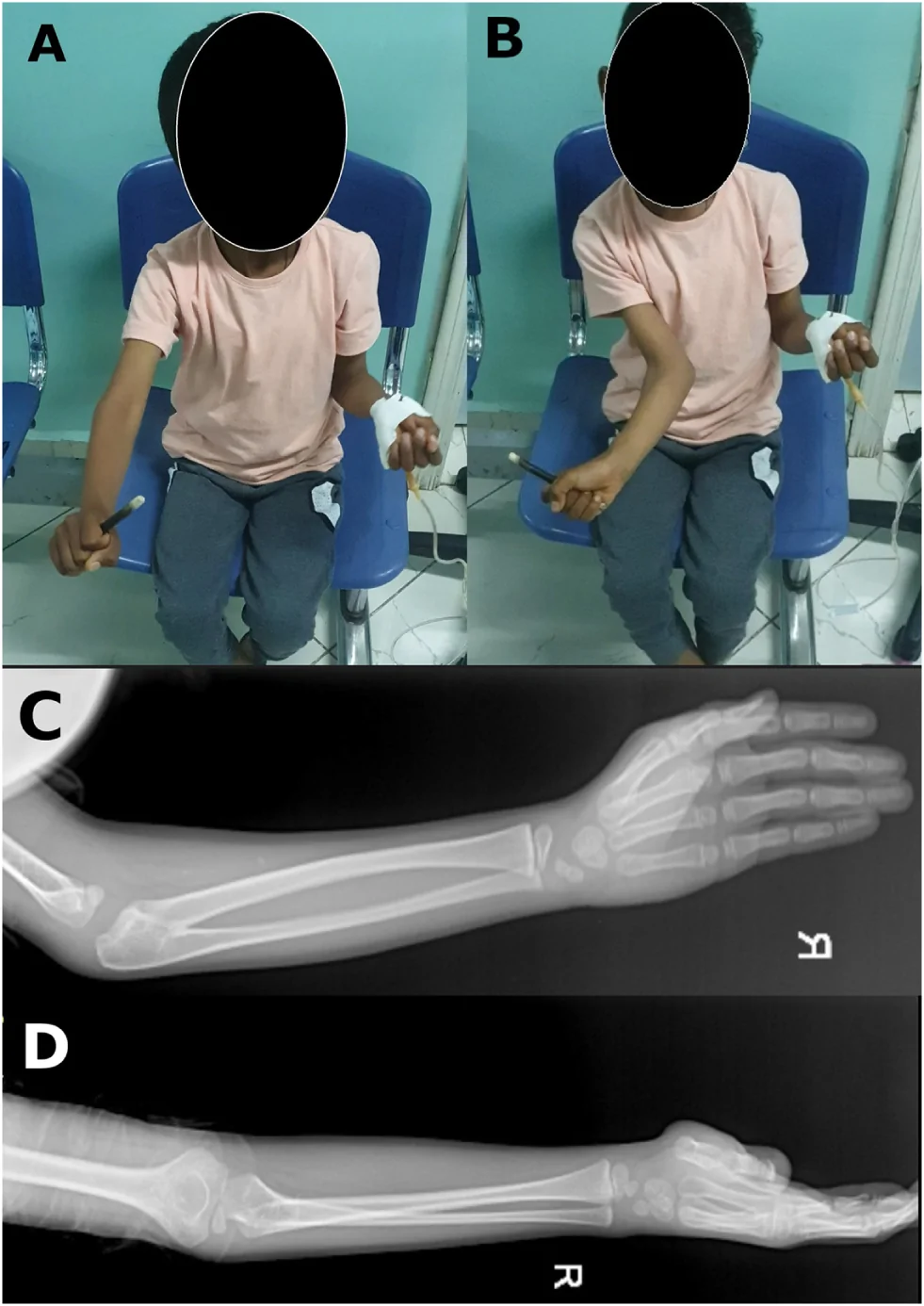
Preoperative findings. (A) Clinical view demonstrating the right forearm locked in fixed pronation. (B) Attempted supination revealing compensatory shoulder adduction and external rotation. (C) Anteroposterior radiograph showing osseous fusion at the proximal radioulnar junction. (D) Lateral radiograph demonstrating an anteriorly dislocated, mushroom-shaped radial head consistent with Cleary-Omer Type IV.

Examination revealed an otherwise healthy child with the right forearm locked in significant pronation (Fig. 1A,B). Attempts at supination triggered compensatory shoulder adduction and external rotation. No additional musculoskeletal anomalies were present, and neurovascular status was intact. The differential diagnosis included isolated congenital radial head dislocation, arthrogryposis, and spastic forearm pronation secondary to an underlying neurological condition; however, fixed, painless pronation present since birth, combined with characteristic radiographic features, established the diagnosis. Radiographs demonstrated osseous fusion at the proximal radioulnar junction and an anteriorly dislocated, malformed radial head, consistent with Cleary-Omer Type IV (Fig. 1C,D). No relevant family history was present, and no drug allergies were documented. Laboratory investigations were unremarkable. Written informed consent was obtained from the patient’s parents.

On 5 September 2023, a derotational osteotomy was performed under general anesthesia at a satellite surgical facility. The patient was positioned supine with the affected limb extended on a radiolucent hand table; no tourniquet was used. The operative field was prepared with a 10% povidone-iodine solution, and ceftriaxone (50 mg/kg) was administered intravenously 30 minutes prior to skin incision. Through a single dorsal incision, the synostosis was exposed via subperiosteal dissection. Under fluoroscopic guidance, a transverse osteotomy was made through the synostosis site. For the dominant right hand, neutral rotation (0°) was selected as the correction target, consistent with established recommendations identifying neutral to slight supination as the functionally optimal forearm position for dominant-hand activities such as writing and self-care^[^8,9^]^. The distal fragment was accordingly rotated from a fixed 70° pronation to neutral, secured with two crossed 2.0-mm Kirschner wires, and a long-arm posterior splint was applied. Operative time was 75 minutes with minimal blood loss.

**Figure 2.**
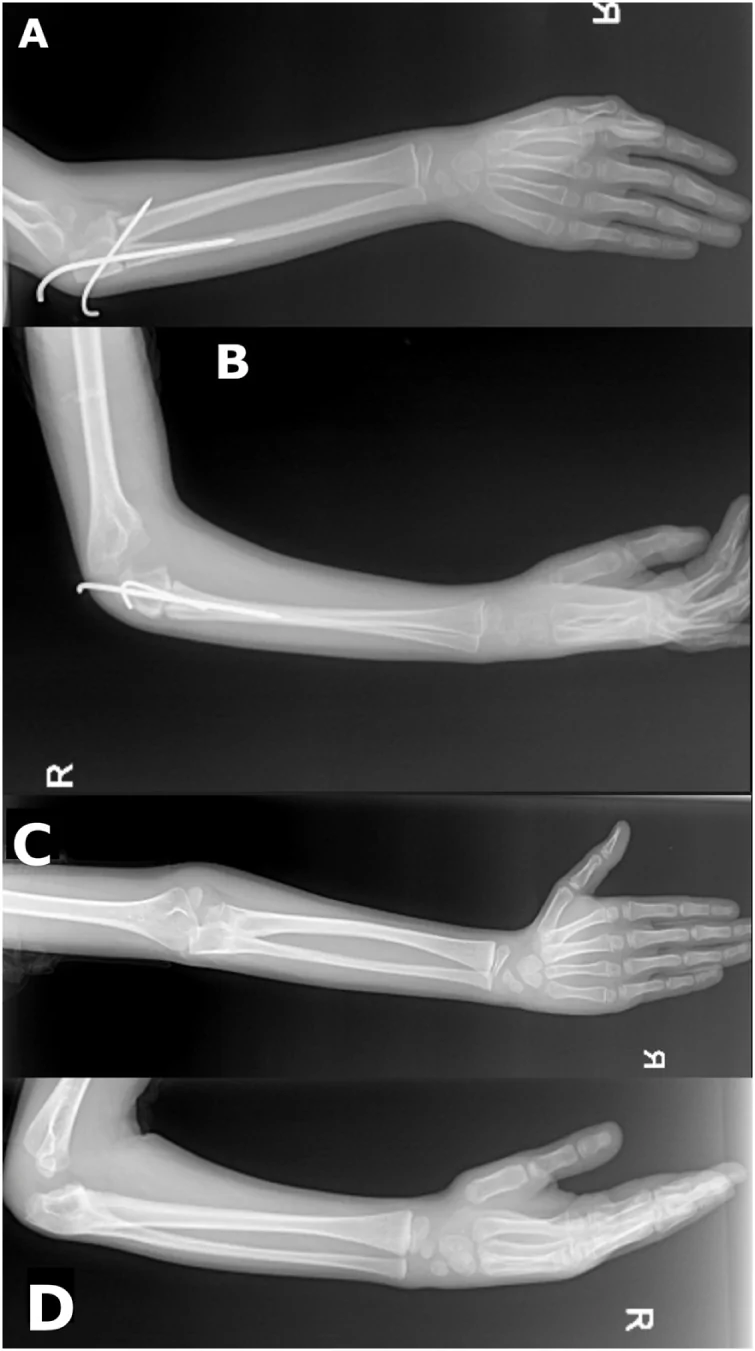
Postoperative radiographic findings. (A) Anteroposterior view at 4 weeks, demonstrating maintained correction with crossed Kirschner wires in situ and early callus formation at the osteotomy site. (B) Lateral view at 4 weeks, confirming satisfactory alignment. (C) Anteroposterior follow-up radiograph showing progressive union. (D) Lateral follow-up radiograph demonstrating complete radiographic union with satisfactory alignment.

**Figure 3.**
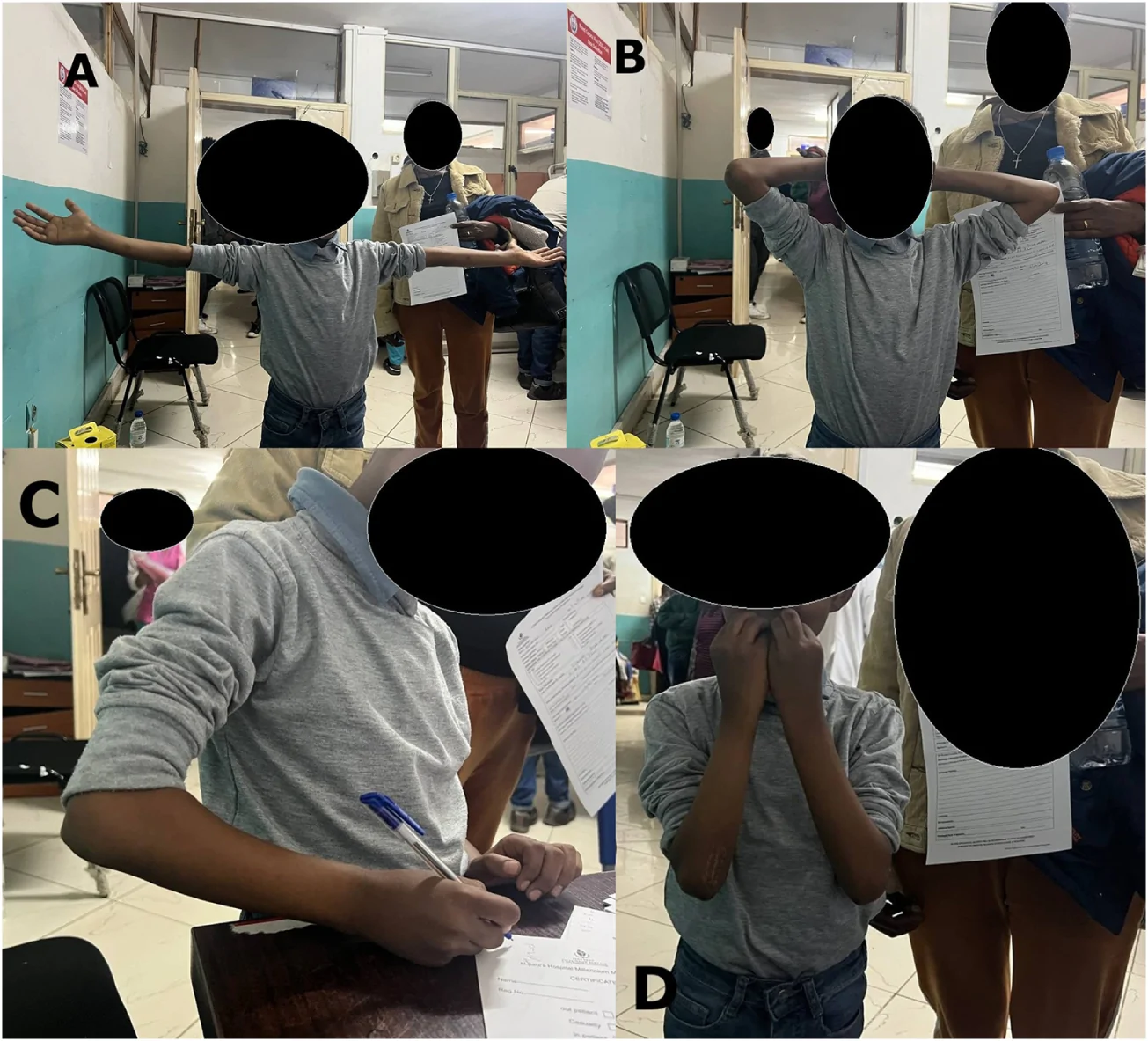
Six-month postoperative clinical photographs demonstrating an excellent functional outcome. (A) Near-full supination achieved without compensatory shoulder movement. (B) Patient demonstrating ability to clasp hands behind the head. (C) Active use of the affected limb for writing. (D) Patient demonstrating the ability to eat independently without difficulty.

Postoperative recovery was uneventful. Kirschner wires were removed at 4 weeks (Fig. 2A,B), after which home-based range-of-motion exercises were progressively introduced; total immobilization also concluded at 4 weeks. Radiographs at 1 and 6 months documented progressive union (Fig. 2C,D). At 6-month follow-up, the forearm rested at neutral (0°), with an active rotational arc from 10° pronation to 60° supination (70° total) and no compensatory shoulder movement (Fig. 3). The child independently fed himself, wrote comfortably, clasped his hands behind his head, and washed without assistance. His mother noted: “He can now feed himself, wash his face, and bathe without any difficulty.” Over a total follow-up of 2 years, no complications were encountered (Table 1).

**Table 2:** Selected published reports and surgical series of congenital proximal radioulnar synostosis.

Author (year)	Country	*n*	Age (year)	Classification	Preop deformity	Intervention	Key outcome
Iyoko IK et al^[^5^]^. (2020)	Nigeria	1	4	Type IV	Not reported	Conservative (non-surgical)	Radiological documentation only; no surgical outcome reported
Edzie EKM et al^[^6^]^. (2023)	Ghana	1	4	Not specified	Not reported	Conservative (non-surgical)	Radiological documentation only; no surgical outcome reported
Barik S et al^[^13^]^. (2021)	Systematic review	Multiple	Pediatric	Mixed	Mean ~ 65° pronation	Derotational osteotomy (various techniques)	Mean improvement to near-neutral; complication rate ~17–18%
Nema SK et al^[^14^]^. (2022)	Systematic review/meta-analysis	Multiple	Pediatric	Mixed	Variable	Corrective derotation osteotomy	Functional improvement across cohorts; complications documented
Present case (2023)	Ethiopia	1	7	Type IV	70° pronation	Derotational osteotomy at synostosis site; crossed 2.0-mm K-wire fixation	Neutral resting position; 10° pronation to 60° supination arc; no complications at 2-year follow-up

**Table 1:** Timeline of key events.

Date	Clinical event
2016 (birth)	Fixed right forearm pronation first noticed during infancy
2016–2023	Progressive functional limitations: difficulty with self-feeding, facial washing, and writing; compensatory shoulder and trunk movements adopted
2023 (prior to September)	First clinic presentation at the treating institution; workup confirming Cleary-Omer Type IV synostosis
5 September 2023	Derotational osteotomy performed under general anesthesia at a satellite surgical facility; fixed 70° pronation corrected to neutral; crossed K-wire fixation; long-arm splint applied
October 2023 (4 weeks post-op)	Kirschner-wire removal; home-based range-of-motion exercises initiated
March 2024 (6 months post-op)	Forearm resting at neutral (0°); active rotational arc 10° pronation to 60° supination; full functional independence confirmed; progressive union on radiograph
September 2025 (2 years post-op)	Final follow-up; complete radiographic union; no complications

## Discussion

Functional impairment from radioulnar synostosis correlates with the degree of rotational fixation, and severe pronation substantially worsens with the educational demands of school age^[^3,10^]^. Early attempts at synostosis resection with interposition materials produced disappointing results owing to high re-synostosis rates^[^11,12^]^. Derotational osteotomy consequently became the established treatment approach.

Published systematic reviews document a mean deformity improvement from approximately 65° of pronation to near-neutral positioning, with complication rates of approximately 17–18%^[^13,14^]^. Unlike the two previously published African case reports, both of which described the condition radiologically without surgical intervention^[^5,6^]^, the present case extends the regional literature by documenting a complete surgical course with two-year follow-up outcomes (Table 2). Our single transverse osteotomy through the synostosis site, with crossed Kirschner-wire fixation, represents the most technically accessible option, avoiding the cost of plate-and-screw constructs while providing adequate stability. Simcock *et al* emphasized that meticulous subperiosteal elevation and controlled osteotomy technique are fundamental to minimizing complications^[^15^]^.

Limitations of this case report include the single-case design, the absence of a validated functional outcome instrument (such as QuickDASH or PODCI), and a two-year follow-up period that may be insufficient to detect late complications such as angular deformity recurrence and re-synostosis. Future African series incorporating standardized functional outcome measures would considerably strengthen the evidence base.

## Conclusion

Derotational osteotomy with Kirschner-wire fixation proved effective and feasible for congenital proximal radioulnar synostosis, yielding meaningful functional improvement in this patient. This first documented surgically treated case from Ethiopia demonstrates that excellent outcomes are achievable in resource-constrained settings. Early identification and timely referral can meaningfully transform the daily lives of affected children.

## Data Availability

All data supporting the findings of this case report are included within the manuscript. Additional clinical records are available from the corresponding authors upon reasonable request.
